# CrossIsoFun: predicting isoform functions using the integration of multi-omics data

**DOI:** 10.1093/bioinformatics/btae742

**Published:** 2024-12-16

**Authors:** Yiwei Liu, Hong-Dong Li, Jianxin Wang

**Affiliations:** School of Computer Science and Engineering, Central South University, Changsha, Hunan 410083, P.R. China; Hunan Provincial Key Lab on Bioinformatics, Central South University, Changsha, Hunan 410083, P.R. China; School of Computer Science and Engineering, Central South University, Changsha, Hunan 410083, P.R. China; Hunan Provincial Key Lab on Bioinformatics, Central South University, Changsha, Hunan 410083, P.R. China; School of Computer Science and Engineering, Central South University, Changsha, Hunan 410083, P.R. China; Hunan Provincial Key Lab on Bioinformatics, Central South University, Changsha, Hunan 410083, P.R. China

## Abstract

**Motivation:**

Isoforms spliced from the same gene may carry distinct biological functions. Therefore, annotating functions at the isoform level provides valuable insights into the functional diversity of genomes. Since experimental approaches for determining isoform functions are time- and cost-demanding, computational methods have been proposed. In this case, multi-omics data integration helps enhance the model performance, providing complementary insights for isoform functions. However, current methods underperform in leveraging diverse omics data, primarily due to the limited power to integrate the heterogeneous feature domains. Besides, among the multi-omics data, isoform-isoform interactions (IIIs) are a key data source, as isoforms interact with each other to perform functions. Unfortunately, IIIs remain largely underutilized in isoform function predictions until now.

**Results:**

We introduce CrossIsoFun, a multi-omics data analysis framework for isoform function prediction. CrossIsoFun combines omics-specific and cross-omics learning for data integration and function prediction. In detail, CrossIsoFun uses a graph convolutional network (GCN) as the omics-specific classifier for each data source. The initial label predictions from GCNs are forwarded to the View Correlation Discovery Network (VCDN) and processed as a cross-omics integrative representation. The representation is then used to produce final predictions of isoform functions. In addition, an antoencoder within a cycle-consistency generative adversarial network (cycleGAN) is designed to generate IIIs from PPIs and thereby enrich the interactomics data. Our method outperforms the state-of-the-art methods on three tissue-naive datasets and 15 tissue-specific datasets with mRNA expression, sequence, and PPI data. The prediction of CrossIsoFun is further validated by its consistency with subcellular localization and isoform-level annotations with literature support.

**Availability and implementation:**

CrossIsoFun is freely available at https://github.com/genemine/CrossIsoFun.

## 1 Introduction

Alternative Splicing (AS) is a crucial regulatory mechanism common in eukaryotes. During this process, different exons of a single gene are combined to produce multiple protein isoforms with different functions (David and Manley 2008, Wu *et al.* 2023). Notably, isoforms play a key role in gene function differentiation (Pan *et al.* 2008, Liu *et al.* 2023). Therefore, accurate annotation of isoform functions is crucial for understanding gene regulation and biological processes.

Nevertheless, knowledge about isoform functions is minimal so far. Meanwhile, traditional experimental technologies are usually time-consuming and costly, leaving a majority of isoforms functionally unannotated. In this case, several computational methods have been proposed to predict isoform functions utilizing functional annotations from the Gene Ontology (GO) database (Gene Ontology Consortium 2004). For example, mi-SVM (Eksi *et al.* 2013), iMILP (Li *et al.* 2014), WLRM (Luo *et al.* 2017), DeepIsoFun (Shaw *et al.* 2019), IsoResolve (Li *et al.* 2021), and IsoFrog (Liu *et al.* 2023) use the expression profiles as the primary input and predict isoform functions with the techniques like multiple instance learning (MIL) (Maron and Lozano-Pérez 1997) and domain adaptation (DA) (Pan *et al.* 2011, Tzeng *et al.* 2014). isopretEM (Karlebach *et al.* 2023) is an expectation-maximization method for inferring isoform-specific functions from the relationships between sequence similarity and functional similarity. However, these methods are limited in that they infer functions solely based on a single data type, ignoring the complementary information that other data types might carry.

Therefore, methods leveraging diverse omics data are developed. IsoFun (Yu *et al.* 2020) propagates labels on a heterogeneous network that incorporates isoform co-expressions, PPIs, and GO hierarchy. DisoFun (Wang *et al.* 2020) also leverages these data to infer isoform functions with collaborative matrix factorization. DIFFUSE (Chen *et al.* 2019) applies a Deep Neural Network (DNN) to predict isoform functions from protein sequences, along with a Conditional Random Field (CRF) built on expression data to refine the predictions. FINER (Chen *et al.* 2021) enhances the DIFFUSE framework by adding PPI data and an III refinement module. DMIL-IsoFun (Yu *et al.* 2021a,b) uses a MIL Convolutional Neural Network (CNN) to initialize isoform annotations from sequence and gene-level annotations and a GCN to refine the annotations with co-expression networks. IsofunGO (Qiu *et al.* 2022) uses a hierarchical network to model GO terms, followed by an attention-based MIL network to fuse multi-omics data and predict isoform functions.

However, their performance is not yet satisfactory, potentially attributed to the limitations in their data fusion strategies. These methods either directly concatenate features from different omics in low-level feature space or process them in the different modules/stages of training, without considering the inter-omics correlations. This might introduce a bias toward specific omics data. Moreover, different types of omics data can present unique characteristics at the high-level label space. Therefore, leveraging the higher-level cross-omics correlations in the label space may help enhance learning performance. Besides the features of individual isoforms, isoform-isoform interactions (IIIs) can also provide insights into the functional roles of isoforms. III refers to the relationships between different isoforms of genes and plays a crucial role in understanding isoform functions. Isoforms often interact and collaborate to execute specific biological functions. However, the shortage of comprehensive III data limits its use in isoform function prediction.

To address these challenges, we introduce CrossIsoFun, a multi-omics data integration framework for isoform function prediction. It generates III data for isoform function prediction and combines omics-specific learning with multi-omics integrative classification within the label space. Specifically, first, we utilize an autoencoder trained with the Cycle-Consistency Generative Adversarial Network (CycleGAN) framework (Wang *et al.* 2021a) to generate IIIs from expression profiles, sequence features, and PPIs. CycleGAN is a type of GAN originally used for unpaired image-to-image translation. Here it allows learning a mapping between different domains (III, expression, and sequence data) without requiring paired data for training. Then, we use the Graph Convolutional Networks (GCNs) (Kipf and Welling 2017) for omics-specific learning to derive initial predictions from expression, sequence, and III data, respectively. Finally, we apply the View Correlation Discovery Network (VCDN) (Wang *et al.* 2021b), a deep learning framework designed to learn and utilize correlations among multiple data views or modalities. In our work, it leverages initial predicted label distributions for cross-omics integration. This strategy facilitates effective multi-omics integration and final function prediction. We show that CrossIsoFun outperforms other state-of-the-art methods on three tissue-naive datasets, which contain samples across different tissues, and 15 tissue-specific datasets with samples and functions associated with a single tissue. Further in-depth analysis reveals that CrossIsoFun’s predictions are consistent with the subcellular localization of isoforms. These observations highlight its effectiveness and reliability in isoform function prediction.

## 2 Materials and methods

### 2.1 Data collection and preprocessing

We apply CrossIsoFun to three tissue-naive datasets and 15 tissue-specific datasets. The tissue-naive datasets include samples from different tissues and conditions, featuring non-tissue-specific functions. In contrast, tissue-specific datasets contain multi-omics data and functions exclusively relevant to certain tissues. The multi-omics data include PPIs (Interactomics), isoform expression profiles from RNA-seq data (Transcriptomics), and sequence features from protein sequences (Proteomics) and conserved domains (Proteomics). Gene-level functional annotations are referenced as labels.

#### 2.1.1 Tissue-naive datasets

Dataset A contains the omics data for 39 375 isoforms from 19 303 human genes, of which 10 271 are single-isoform genes (SIGs) and 9032 are multi-isoform genes (MIGs). The expression profiles are composed of 1735 RNA-seq runs (Chen *et al.* 2019) from the NCBI Sequence Read Archive database [SRA (Leinonen *et al.* 2010)] and quantified in Transcripts Per Million (TPM). Sequence features are derived from protein sequences and conserved domains. Protein sequences are translated from the “Coding DNA Sequence” (CDS) from NCBI Reference Sequences [RefSeq, GRCh38.p14 (Pruitt *et al.* 2012)]. Then we feed them into ProtTrans (Elnaggar *et al.* 2022) to obtain their embedding; Conserved domains for each isoform are acquired from the NCBI Conserved Domain Database [CDD (Marchler-Bauer *et al.* 2015)] and processed as a 64D feature vector, referencing (Chen *et al.* 2019). The sequence feature vector is generated by concatenating the conserved domain feature and protein sequence embedding. For PPI data, we collect 6 761 370 physical PPIs with experimental supports from eight well-curated PPI databases (see Supplementary Table S1 for details). The PPIs are then directly assigned to the isoforms of the gene nodes.

The other two tissue-naive datasets (called Dataset B and Dataset C) are obtained from the literature (Eksi *et al.* 2013) and (Li *et al.* 2014), respectively. The details for data collection and preprocessing are given in Supplementary Note S1.

The labels for training and testing of our model are obtained from gene functional annotations in the 11 June 2023 version of the Gene Ontology (GO) knowledgebase. Specifically, we use GO slim terms (Shaw *et al.* 2019) in our experiment, which are selected as they represent expert-curated, high-level functional categories in the Gene Ontology Annotation (GOA) project (Ashburner *et al.* 2000). These terms cover key aspects of Cellular Component, Biological Process, and Molecular Function, providing a concise yet comprehensive basis for comparative evaluations. This approach aligns with prior studies, such as DeepIsoFun, DIFFUSE, IsoResolve, and IsoFrog, which also utilized these terms for benchmarking. Following these methods, we filter the GO slim terms by removing the GO terms that represent too specific (size < 5) or too general (size > 1000) functions and finally obtain 96 GO slim terms to evaluate model performance.

#### 2.1.2 Tissue-specific datasets

The tissue-specific datasets are obtained from Chen *et al.* (2021), which includes 12 datasets for major human tissues and three for brain sub-tissues. These tissues are recorded in the BRENDA Tissue Ontology and associated with multiple valid tissue-specific GO terms, which specifically describe the cellular functions of the tissues (Greene *et al.* 2015).

RNA-seq data for the tissues are sourced from the NCBI SRA database according to the accession numbers from Greene *et al.* (2015) and Razmara *et al.* (2019). The methodology for acquiring sequence features is identical to that in Section 2.1.1, retaining 43 289 isoforms from 19 408 genes. PPI data including 317 750 physical interactions among the 19 408 genes is collected from Zitnik and Leskovec (2017). Since the way that genes interact might vary greatly across different tissues, tissue-specific PPI networks are constructed. Referencing (Chen *et al.* 2021), genes that exhibit high specificity to certain tissues are selected, i.e. the “tissue-enhanced genes.” For each major tissue, a gene is defined as tissue-enhanced if its expression is at least 4-fold over its average across other major tissues. For brain tissues, the threshold is set as 2-fold. Then the tissue-specific network is extracted from the global PPI network by maintaining the connections between nodes in which at least one is tissue-enhanced.

### 2.2 Method

Sequence, expression profiles, and III data can reflect the function roles of isoforms. Expression profile aids in isoform function prediction by providing detailed information about the expression levels and splicing patterns of isoforms in different tissues or conditions and hence reflect their functions. Sequence data is a key resource for predicting isoform functions since it captures the specific amino acid composition and structural features that define each isoform. Differences in sequence can lead to variations in protein domains, motifs, and active sites, directly influencing the isoform’s function. The rationale of applying III data in isoform function prediction lies in that isoforms execute specific functions by interacting with other isoforms, hence analyzing these interactions can reveal valuable insights into isoform functions. In this case, integrating these data types allows us to exploit the complementary functional information they contain, thereby enhancing the prediction of isoform functions.

Therefore, the primary objective of the model architecture is to integrate the three data types for isoform function prediction. Briefly, we first use a CycleGAN-like architecture to generate III data from expression, sequence, and PPI data due to the shortage of comprehensive III data. Next, we use a GCNs + VCDN architecture to integrate the multi-omics data for function prediction.

**Figure 1.**
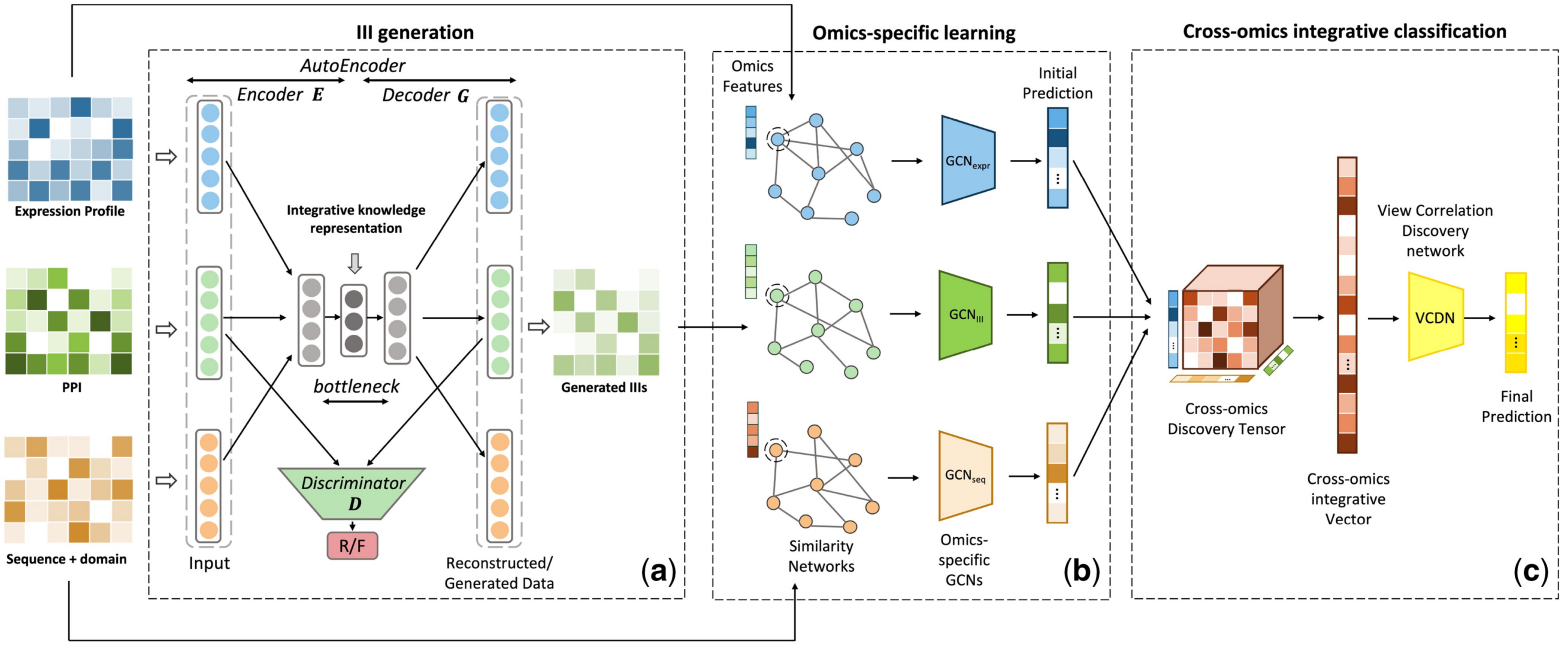
Schematic of CrossIsoFun. (a) The III generation framework. (b) The omics-specific GCNs for initial prediction. (c) The VCDN for multi-omics data integration and isoform function prediction.

The workflow of CrossIsoFun is illustrated in Fig. 1. It includes three components, which will be described in detail in the following sections.

#### 2.2.1 Notations

The expression profiles are represented as a feature matrix $X^{\left(1\right)}\in R^{n\times e}$, where n denotes the number of isoforms, and e represents the number of RNA-seq experiments included in the dataset. Batch effects typically arise due to differences in experimental conditions or batches across study samples. Since RNA-Seq samples are used as feature columns in our matrix $X^{\left(1\right)}$, batch effects do not impact the predictions in this study. The sequence features of all the isoforms are stored in a matrix $X^{\left(2\right)}\in R^{n\times s}$, where s = 1088. PPIs are encoded as a binary matrix $X^{\left(3\right)}\in R^{n\times g}$, where g denotes the number of genes. Specifically, in the matrix representing PPI data ($X^{\left(3\right)}$), each row corresponds to an isoform and each column corresponds to a gene. The entries in this matrix are defined as follows: $X_{i,m}^{\left(3\right)}=1$ indicates that the gene producing isoform i interacts with gene m in the PPI network; $X_{i,m}^{\left(3\right)}=0$ indicates no interaction between gene m and the gene producing isoform i. Since an isoform might be annotated with multiple GO terms, we regard isoform function prediction as a multi-label learning problem. Therefore, gene labels are stored in a binary matrix $Y\in R^{g\times T}$. Specifically, T means the number of GO terms. For a given GO term, if a gene is annotated within it, the gene is tagged with 1, otherwise with 0.

#### 2.2.2 III generation framework

III data remains largely underutilized in isoform function predictions due to its incompleteness. However, the inherent biological properties of isoforms enable the computational inference and interpolation of missing IIIs using data such as expression, sequences, and PPIs. Expression profiles reflect co-expression trends, sequence features determine structural and functional domains, and PPI data offers a direct biological context for isoform interactions. Therefore, we design an autoencoder trained within a cycleGAN framework to infer III data from these data. This framework comprises two principal components: an autoencoder network and a discriminator network (D) (Fig. 1a).

**(1) Autoencoder**

The autoencoder network is designed as a multi-input and multi-output architecture. It is composed of an encoder (E) and a decoder (G). The encoder E is a stacked fully connected network with three input layers for expression, sequence, and PPI data, respectively. The inputs are then channeled through the shared interlayers and converged at the bottleneck to form an integrative knowledge representation. A decoder G structured symmetrically to the encoder is used to reconstruct the inputs and generate the missing data from the integrative representation. Notably, the dimension of the output layer corresponding to PPIs is adjusted to match that of the IIIs, thus facilitating the generation of III data. All parameters except those in the input and output layers are shared across different data types, ensuring the generation of a specific data type even when it is absent in the inputs.

The primary training objective for the autoencoder is to generate III data for each isoform using its expression profile, sequence feature, and PPI vector. Reconstruction of the inputs is also required. We denote the input feature vector for the i th isoform as $x_{i}^{\left(k\right)}$ (the i th row of $X^{\left(k\right)}$), the whole generation/reconstruction output as $O^{\left(k\right)}$ and the output vector of the i th isoform as $o_{i}^{\left(k\right)}$, where k = 1, 2, 3 for expression, sequence, and PPI data, respectively. Then we have the generation and reconstruction losses below:

(1) $$\begin{array}{c} L_{\text{GEN}}=L_{\text{BCE}}(X^{\left(3\right)},O^{\left(3\right)})=\sum_{i=1}^{n}L_{\text{BCE}}(x_{i}^{\left(3\right)},o_{i}^{\left(3\right)})\\ =\sum_{i=1}^{n}L_{\text{BCE}}(x_{i}^{\left(3\right)},G^{\left(3\right)}(E(x_{i}^{\left(1\right)},x_{i}^{\left(2\right)},x_{i}^{\left(3\right)}))), \end{array}$$

(2) $$\begin{array}{c} L_{\text{REC}}=\sum_{k=1}^{2}L_{\text{MSE}}(X^{\left(k\right)},O^{\left(k\right)})=\sum_{k=1}^{2}\sum_{i=1}^{n}L_{\text{MSE}}(x_{i}^{\left(k\right)},o_{i}^{\left(k\right)})\\ =\sum_{k=1}^{2}\sum_{i=1}^{n}L_{\text{MSE}}(x_{i}^{\left(k\right)},G^{\left(k\right)}(E(x_{i}^{\left(1\right)},x_{i}^{\left(2\right)},x_{i}^{\left(3\right)}))), \end{array}$$

where $G^{\left(k\right)}$ represents the decoding process corresponding to the data type k. We use the Binary Cross-Entropy (BCE) loss for the PPI data as it is represented in binary format. For any given PPI, at least one pair of isoforms from the respective genes must interact. Therefore, for each gene pair, we choose the maximum generated value among its isoform pairs to compare against the actual value indicating the relationship between the gene pair. This ensures that the generated III results strictly follow the context of PPIs. The Mean Squared Error (MSE) loss is applied to other data types as in the training norms of GANs. Besides, we suppose all the generated and reconstructed data can be used for further generation and reconstruction via the autoencoder, which is the “cycle consistency” part of the training scheme. L1 loss is applied in the cycle consistency loss, which is detailed below:

(3) $$\begin{array}{c} L_{\text{cycle}}=\sum_{k=1}^{3}\sum_{i=1}^{n}L1(x_{i}^{\left(k\right)},G^{\left(k\right)}(E(o_{i}^{\left(1\right)},o_{i}^{\left(2\right)},o_{i}^{\left(3\right)})))). \end{array}$$

Therefore, the total loss for the primary training objective is:

(4) $$\begin{array}{c} L_{AE}=\lambda_{1}L_{\text{GEN}}+\lambda_{2}L_{\text{REC}}+\lambda_{3}L_{\text{cycle}}, \end{array}$$

where $\lambda_{1}$, $\lambda_{2}$, and $\lambda_{3}$ are the parameters adjusting the impact of each term in the function.

We also establish three auxiliary training objectives: generating expression profiles from sequence features and PPIs, generating sequence features from expression profiles and PPIs, generating IIIs from expression profiles and sequence features. By capturing correlations across diverse omics data, this auxiliary training scheme strengthens the module’s ability to integrate complementary information. This ultimately improves the performance of the III generation module in isoform function prediction. The losses for the auxiliary generation objectives, denoted as $L_{AE}^{\left(1\right)}$, $L_{AE}^{\left(2\right)}$, and $L_{AE}^{\left(3\right)}$, are similar to the one for the primary objective and detailed in Supplementary Note S2. Then the total loss for training the autoencoder can be calculated as

(5) $$\begin{array}{c} L_{AE(\mathrm{total})}=L_{AE}+L_{AE}^{\left(1\right)}+L_{AE}^{\left(2\right)}+L_{AE}^{\left(3\right)}. \end{array}$$

**(2) Discriminator**

Since our goal is III generation, adversarial training is specifically performed on the III generation process. The discriminator D is composed of three stacked fully connected layers and linked to the III output layer, differentiating between the generated data and the actual PPI inputs. Therefore, the adversarial loss for the framework is

(6) $$\begin{array}{c} L_{\text{GAN}}(G,D)=\sum_{i=1}^{n}\underset{G}{\min}\underset{D}{\max}E_{x∼P_{X^{\left(3\right)}}}[\text{log}\;D(x)]+\\ \;\text{log}(1-D(o_{i}^{\left(3\right)})). \end{array}$$

III data is derived by feeding $X^{\left(1\right)}$, $X^{\left(2\right)}$, and $X^{\left(3\right)}$ into the trained autoencoder $G^{\left(3\right)}(E(\cdot ))$. The generated data is then processed as a symmetrical adjacency matrix to serve as the interactomics input ${\tilde{X}}^{\left(3\right)}$ for function prediction.

(7) $$\begin{array}{c} {\tilde{X}}^{\left(3\right)}=\mathrm{symm}(G^{\left(3\right)}(E(X^{\left(1\right)},X^{\left(2\right)},X^{\left(3\right)}))). \end{array}$$

The process of symmetrization is detailed in Supplementary Note S3.

#### 2.2.3 GCNs for omics-specific learning and initial label prediction

We use GCNs for omics-specific learning, training a GCN on each omics data to make an initial label prediction (Fig. 1b).

First, a weighted network is built referencing (Wang *et al.* 2021b) to serve as the graph input for GCN. Denoting the feature matrix by $X\in R^{n\times d}$ (d is the feature dimensionality) and the feature vector of the ith sample by $x_{i}$, the network can be modeled as an adjacency matrix $A\in R^{n\times n}$ where

(8) $$\begin{array}{c} A_{ij}=\{\begin{array}{cc} s_{ij}, & \text{if}\;s_{ij}\geq \theta \;\text{and}\;i\neq j,\\ 0, & \text{otherwise}, \end{array} \end{array}$$

$s_{ij}$ here is the cosine similarity between $x_{i}$ and $x_{j}$. The threshold θ is set as the (n×a)th largest value of $s_{ij}$. Here a denotes the pre-defined average number of edges retained for each node.

Then, we construct a GCN with three convolutional layers. Taking X and A as inputs, each convolutional layer is formulated as:

(9) $$\begin{array}{c} H^{l+1}=f\left(H^{l},\tilde{A}\right)=\sigma^{l}\left(\tilde{A}H^{l}W^{l}\right), \end{array}$$

where $H^{l}$ represents the input for the lth convolutional layer of the GCN and $H^{1}=X$. $W^{l}$ represents the weight matrix corresponding to the layer and $\sigma^{l}\left(\cdot \right)$ denotes its nonlinear activation function. $\tilde{A}\in R^{n\times n}$ is calculated based on A as follows:

(10) $$\begin{array}{c} \tilde{A}={\bar{P}}^{-\frac{1}{2}}\bar{A}{\bar{P}}^{-\frac{1}{2}}={\bar{P}}^{-\frac{1}{2}}(A+I){\bar{P}}^{-\frac{1}{2}}, \end{array}$$

where $\bar{P}$ is the degree matrix of $\bar{A}$ and I is an identity matrix with the dimensions identical to A. The convolutional layers enable the learning of meaningful representations by considering the features of individual isoforms and the information propagated through the similarity network.

A fully connected layer is stacked after the convolutional layers and used to calculate the initial prediction scores for isoform functions. Specifically, let $H^{L}$ represent the output from the last convolutional layer, the operations within the fully connected layer include:

(11) $$Z=H^{L}W^{L}+b^{L},$$

where $W^{L}$ represents the weights of the fully connected layer, and $b^{L}$ denotes the bias vector. Z then undergoes an element-wise sigmoid activation, providing the probability prediction for each function:

(12) $$\hat{Y}=\mathrm{GCN}(X,A)=\sigma^{L}(Z),$$

where $\sigma^{L}$ is the sigmoid function, and $\hat{Y}\in R^{n\times T}$ represents the prediction result.

A single gene can produce multiple isoforms, each with potentially distinct functions. The overall functional profile of a gene is an aggregation of its isoforms’ functions. For any function annotated to a gene, at least one isoform of that gene is expected to undertake that function. Based on this relationship, we use the isoform with the highest prediction score to represent its gene for each function. We denote the predicted probability distribution of the mth gene across all the functions as ${\hat{y}}_{m}$. Then BCE Loss is used for optimization:

(13) $$L_{\mathrm{GCN}}=\sum_{m=1}^{g}L_{\text{BCE}}(y_{m},{\hat{y}}_{m}),$$

where $y_{m}$ represents the one-hot encoded label vector of the mth gene, derived from the mth row of Y. In addition, to address label imbalance, we assign different weights to the losses for different classes with the weight assignment approach in Wang *et al.* (2021b). Specifically, we use a weighted Binary Cross-Entropy loss (WBCE loss) for each GO term, which can be formulated as:

(14) $$L_{\text{WBCE}}=-\frac{1}{n}\sum_{i=1}^{n}[w_{\text{pos}}\times y_{i}\times \text{log}({\hat{y}}_{i})+w_{\text{neg}}\times (1-y_{i})\times \text{log}(1-{\hat{y}}_{i})],$$

where $w_{\text{pos}}$ is the weight for the positive class, which is set to the inverse of the proportion of positive samples among all genes within the training set. Similarly, $w_{\text{neg}}$ is the weight for the negative class, set to the inverse ratio of negative samples. The function computes the losses for all the training samples simultaneously, guiding the GCN to learn and predict the label distribution effectively.

We then obtain the initial prediction for $X^{\left(k\right)}$ with the trained $GCN^{\left(k\right)}$:

(15) $${\hat{Y}}^{\left(k\right)}=\mathrm{GC}N^{\left(k\right)}(X^{\left(k\right)},A^{\left(k\right)}).$$

The details of training and testing of the GCNs are provided in Supplementary Note S4.

#### 2.2.4 Multi-omics integrative prediction of isoform functions

CrossIsoFun uses a VCDN to capture cross-omics correlations in the label space. This strategy considers the omics-specific insights from the high-level space and hence effectively integrates multi-omics data.

Specifically, we leverage a VCDN to integrate expression profiles, sequence features, and IIIs, and perform the final predictions of isoform functions (Fig. 1c). To predict isoform functions for the ith sample, we first integrate its initial predictions from the GCNs corresponding to all the omics data. In detail, we symbolize the ith row of ${\hat{Y}}^{\left(k\right)}$ as ${\hat{y}}_{i}^{\left(k\right)}$, and obtain a cross-omics discovery tensor $C_{i}\in R^{T\times T\times T}$ with each entry computed as:

(16) $$C_{i,(e_{1},e_{2},e_{3})}={\hat{y}}_{i(e_{1})}^{\left(1\right)}{\hat{y}}_{i(e_{2})}^{\left(2\right)}{\hat{y}}_{i(e_{3})}^{\left(3\right)},$$

where ${\hat{y}}_{i(e)}^{\left(k\right)}$ represents the eth entry of ${\hat{y}}_{i}^{\left(k\right)}$. Then $C_{i}$ is reshaped as a vector $c_{i}\in R^{T^{3}}$ and input into the VCDN, a fully connected network to perform the classification task. We have

(17) $${\hat{y}}_{i}^{\left(\text{VCDN}\right)}=\mathrm{VCDN}(c_{i}),$$

where ${\hat{y}}_{i}^{\left(\text{VCDN}\right)}\in R^{T}$ stores the predicted probabilities of isoform i across all the functions. Similar to the training of GCNs, the highest prediction score among the isoforms of a gene is regarded as the prediction result of the gene. Denoting the scores of the mth gene by ${\hat{y}}_{m}^{\left(\text{VCDN}\right)}$, the loss function for optimization is defined as:

(18) $$L_{\text{VCDN}}=\sum_{m=1}^{g}L_{\text{WBCE}}(y_{m},{\hat{y}}_{m}^{\left(\text{VCDN}\right)}),$$

CrossIsoFun involves the joint training of omics-specific learning and omics integrative classification. We start by pretraining each omics-specific GCN to obtain an optimal initialization. In each following epoch, we first fix the VCDN and update the GCN for each data type. Subsequently, we fix the omics-specific GCNs and update the VCDN. This alternating update process between omics-specific GCNs and the VCDN continues until convergence.

In the training process, the following steps are taken to fine-tune the parameters: (i) Hyperparameter Grid Search: We conduct a systematic grid search over various hyperparameters, such as learning rates, the number of layers, and the number of neurons in the generator and discriminator networks. (ii) Learning Rate Scheduling: Different learning rate schedules are evaluated, with adaptive learning rates in the Adam optimizer yielding the most stable convergence. (iii) Regularization Techniques: Dropout and weight normalization are implemented to mitigate overfitting and mode collapse. Dropout rates are fine-tuned for enhanced performance. (iv) Validation Strategy: A validation set comprising 10% of the training data is used to monitor model performance and dynamically adjust hyperparameters to avoid overfitting or underfitting. (v) Early Stopping: Early stopping based on validation loss is applied to reduce training time and computational overhead.

### 2.3 Implementation

CrossIsoFun is implemented in Python. We have made the source codes publicly available on GitHub (https://github.com/genemine/CrossIsoFun). To ensure ease of use, the repository includes: (i) A README file that provides detailed instructions on input data preparation, implementation steps, software dependencies, and usage guidelines, and (ii) Specific instructions and code snippets for training a model and making predictions on test datasets, enabling users to straightforwardly implement and apply CrossIsoFun. The method relies solely on sequence, expression, and III data as inputs, without requiring preexisting gene-level annotations.

## 3 Performance evaluation

### 3.1 Comparison of CrossIsoFun with state-of-the-art methods

We compare the performance of CrossIsoFun on Dataset A, B, and C with 12 state-of-the-art methods (mi-SVM, iMILP, WLRM, IsoFun, DisoFun, DIFFUSE, IsoResolve, IsoFrog, DMIL-Isofun, FINER, isofunGO, and isopretEM). The parameters for the methods are configured as recommended by the authors or fine-tuned within the suggested ranges. For each dataset, we partition 80% into the training set (10% as the validation set) and 20% into the testing set. To prevent information leakage, the isoforms derived from the same gene are allocated to the same set. Moreover, we avoid the genes from the same homologous group and their isoforms being divided into different sets. The homologous genes are obtained from the Duplicated Genes Database (Ouedraogo *et al.* 2012), and defined based on pairwise BLAST comparisons and genomic locations. The data split is consistent across all evaluated GO terms. To ensure a fair comparison across different methods, we use the following strategies: (i) consistent data splits: all methods are trained and tested on the same dataset, with identical training and test splits. This consistency ensures that performance differences reflect model capabilities rather than variations in data handling. (ii) Standardized implementation: we implement all comparison methods using publicly available source code to ensure alignment with their original experimental setups. This approach guarantees valid implementation and consistent evaluation criteria. In addition, in the experiment, we do not use the SwissProt data and corresponding labels for pretraining DIFFUSE and FINER as done in Chen *et al.* (2019) and Chen *et al.* (2021). The hyperparameters for CrossIsoFun are tuned experimentally or set as recommended in Wang *et al.* (2021a,b), which are detailed in Supplementary Table S2.

Model performance is evaluated using the median AUC and AUPRC values across the GO slim terms. To check the consistency between the predictions and gene annotations for each GO term, the highest prediction score among the isoforms of each gene is used. The results are detailed in Table 1. CrossIsoFun achieves superior performance over the compared methods across all the datasets. The AUCs of CrossIsoFun on Dataset A, B, and C are 0.884, 0.949, and 0.865, respectively, all significantly exceeding the results from the other methods in comparison (*P*-value < 0.05). For Dataset A, CrossIsoFun gains improvements over mi-SVM, iMILP, WLRM, IsoFun, DisoFun, IsoResolve, IsoFrog, DIFFUSE, DMIL-Isofun, FINER, IsofunGO, and isopretEM by 280.2%, 317.4%, 138.5%, 117.0%, 151.0%, 79.4%, 56.7%, 72.2%, 46.0%, 27.2%, 18.5%, and 20.4% respectively (against the baseline of 0.5). For Dataset B, the improvements are 199.3%, 242.8%, 256.4%, 131.4%, 223.0%, 52.2%, 28.3%, 43.9%, 29.8%, 14.5%, 19.1%, and 14.8% respectively, while the improvements are 350.6%, 168.4%, 214.7%, 99.5%, 199.2%, 78.9%, 53.4%, 59.4%, 39.8%, 29.0%, 14.8%, and 16.6% for Dataset C. AUPRC comparisons further demonstrate the performance advancement of CrossIsoFun over the other methods. For instance, CrossIsoFun shows an improvement of 579.0%, 911.8%, 588.0%, 606.9%, 570.1%, 263.3%, 216.6%, 196.6%, 155.4%, 59.8% 28.4%, and 36.5% in AUPRC on Dataset A when compared with mi-SVM, iMILP, WLRM, IsoFun, DisoFun, IsoResolve, IsoFrog, DIFFUSE, DMIL-Isofun, FINER, IsofunGO, and isopretEM, respectively (against the baseline of 0.1).

**Table 1.** The performance of CrossIsoFun and state-of-the-art methods on tissue-naive datasets in terms of the median AUC and AUPRC for the 96 GO slim terms.^a^

Method	Dataset A		Dataset B		Dataset C	
	AUC	AUPRC	AUC	AUPRC	AUC	AUPRC
**CrossIsoFun**	**0.884**	**0.616**	**0.949**	**0.637**	**0.865**	**0.539**
isopretEM	0.819 (7.2e−10)	0.478 (1.6e−17)	0.891 (4.4e−04)	0.469 (5.8e−06)	0.813 (7.7e−11)	0.379 (2.1e−21)
IsofunGO	0.824 (5.1e−08)	0.502 (2.1e−12)	0.877 (1.8e−07)	0.456 (7.4e−07)	0.818 (6.3e−10)	0.367 (2.9e−23)
FINER	0.802 (4.4e−13)	0.423 (4.5e−23)	0.892 (9.3e−04)	0.475 (1.2e−06)	0.783 (9.9e−12)	0.321 (1.6e−27)
DMIL-Isofun	0.763 (2.6e−25)	0.302 (3.8e−35)	0.846 (6.2e−09)	0.426 (1.4e−10)	0.761 (3.5e−15)	0.314 (7.3e−29)
DIFFUSE	0.723 (1.7e−35)	0.274 (5.1e−49)	0.812 (1.3e−13)	0.404 (5.0e−11)	0.729 (3.4e−24)	0.245 (8.9e−41)
IsoFrog	0.745 (4.4e−29)	0.263 (5.8e−51)	0.850 (2.5e−08)	0.416 (3.1e−10)	0.738 (5.3e−22)	0.231 (3.7e−43)
IsoResolve	0.714 (4.2e−38)	0.242 (2.6e−54)	0.795 (2.9e−15)	0.367 (1.4e−13)	0.704 (2.2e−30)	0.204 (1.2e−47)
DisoFun	0.653 (7.6e−55)	0.177 (4.1e−62)	0.639 (2.5e−40)	0.190 (2.9e−26)	0.622 (4.5e−50)	0.166 (8.7e−51)
IsoFun	0.677 (1.8e−48)	0.173 (1.1e−66)	0.694 (4.5e−34)	0.213 (3.6e−25)	0.683 (1.5e−35)	0.190 (6.0e−48)
WLRM	0.661 (2.8e−65)	0.175 (2.1e−64)	0.626 (3.3e−45)	0.193 (1.0e−26)	0.616 (2.0e−51)	0.164 (4.3e−54)
iMILP	0.592 (1.1e−69)	0.151 (1.9e−70)	0.631 (1.7e−42)	0.162 (4.5e−29)	0.636 (7.7e−47)	0.189 (4.2e−50)
mi-SVM	0.601 (1.2e−67)	0.176 (1.7e−63)	0.650 (1.0e−38)	0.190 (6.2e−27)	0.581 (4.1e−59)	0.128 (1.2e−59)

a Values in the parentheses are the *P*-values (*t*-test) obtained from statistical comparisons between CrossIsoFun and other methods. Bold values indicate the maximal AUC or AUPRC among different methods.

The notable improvements achieved across multiple human and mouse datasets indicate the effectiveness and robustness of our method. Specifically, CrossIsoFun outperforms iMILP, mi-SVM, WLRM, IsoResolve, and IsoFrog (only input expression data for predictions) by a large margin. This observation supports the assumption that incorporating sequence and interaction data might enhance the predictive capability of the models. The improvement of CrossIsoFun over isopretEM also demonstrates the strength of multi-omics data integration. The performance superiority of CrossIsoFun over IsofunGO, DMIL-IsoFun, FINER, DIFFUSE, and DisoFun highlights the advantage of our strategy to integrate multi-omics data in the label space for exploiting the complementary functional information inherent in different data types.

Model assessment and method comparison are also conducted on the 15 tissue-specific datasets, focusing on the methods that apply multi-omics data for predictions, the newest tissue-specific function prediction method TS-Isofun (Yu *et al.* 2022), and isopretEM. Performance evaluations on GO terms in major tissues (421) and brain tissues (50) are presented in Table 2. As shown in the results, CrossIsoFun outperforms isopretEM and FINER, which show the best performance among the compared methods in the major tissue and brain tissue datasets, respectively. The improvements of CrossIsoFun over isopretEM are about 17.1% and 32.3% in AUC and AUPRC for major tissues, and 25.8% and 44.3% over FINER for brain tissues, respectively. In addition, it can be noted that CrossIsoFun generally shows smaller standard deviations (SDs) across tissues than other methods, indicating its promising ability for function identification under more specific circumstances and further proving its robustness. The performance of CrossIsoFun on GO terms specific to each tissue is given in Supplementary Table S3.

**Table 2.** Comparison of functional prediction performance between CrossIsoFun and methods using multi-omics data on tissue-specific datasets.

Method	Major tissue datasets		Brain tissue datasets	
	AUC (SD)	AUPRC (SD)	AUC (SD)	AUPRC (SD)
DisoFun	0.805 (0.188)	0.460 (0.236)	0.770 (0.214)	0.419 (0.242)
DIFFUSE	0.836 (0.134)	0.568 (0.208)	0.822 (0.190)	0.541 (0.256)
DMIL-Isofun	0.855 (0.172)	0.607 (0.216)	0.839 (0.221)	0.577 (0.242)
FINER	0.894 (0.080)	0.634 (0.158)	0.876 (0.115)	0.655 (0.234)
IsofunGO	0.881 (0.101)	0.635 (0.207)	0.862 (0.175)	0.623 (0.212)
TS-Isofun	0.841 (0.188)	0.551 (0.236)	0.814 (0.214)	0.518 (0.267)
isopretEM	0.903 (0.044)	0.669 (0.143)	0.869 (0.176)	0.647 (0.239)
**CrossIsoFun**	**0.972 **(0.051)	**0.853 **(0.132)	**0.973 **(0.072)	**0.901 **(0.143)

Bold values indicate the maximal AUC or AUPRC among different methods.

### 3.2 Performance on GO terms of different sizes and functional categories

Previous studies (Eksi *et al.* 2013, Shaw *et al.* 2019, Li *et al.* 2021) have indicated that both the size and category of GO terms impact the effectiveness of isoform function prediction. Specifically, the size of a GO term is the number of genes annotated to it, while the category refers to its classification within the three main branches: biological process (BP), cellular component (CC), and molecular function (MF). To examine the impact of these factors on our method, we perform CrossIsoFun on GO terms with different sizes and categories based on Dataset A.

First, 96 GO slim terms are split into four groups, in which the sizes of GO terms range within [10, 150], [151, 300], [301, 600], and [601, 1000], respectively. AUCs and AUPRCs from SIGs and MIGs serve as the metrics for assessing the performance of CrossIsoFun on each GO term. The experiment results are given in Supplementary Fig. S1. The median SIG-level AUCs for the four groups are 0.916, 0.925, 0.860, and 0.833, respectively, while the median AUPRCs on SIGs are 0.716, 0.726, 0.598, and 0.523, respectively. The performance on MIGs exhibits only slight variations from the SIG-level results. Supplementary Fig. S1 reveals a trend of enhanced performance for smaller GO terms, aligning with the findings in previous studies (Li *et al.* 2014, Chen *et al.* 2019, Shaw *et al.* 2019, Li *et al.* 2021, Liu *et al.* 2023). The main reason may be that larger GO terms often represent more generalized functions, making it difficult for models to learn specific patterns. In addition, for larger GO terms, certain functions might be overrepresented, leading to biased training and a lack of generalization in the model.

Then we divide GO slim terms into BP, CC, and MF groups and evaluate the performance of CrossIsoFun within these categories. As shown in Supplementary Fig. S1, CrossIsoFun demonstrates robust performance across different GO branches. For instance, the median AUCs on SIGs for BP, CC, and MF terms are 0.872, 0.855, and 0.926, respectively. This indicates the consistent effectiveness of our method across categories with different functional significance.

### 3.3 Performance on differentiating functions of isoforms with different localization

The locations of isoforms within different subcellular compartments determine the environments where the isoforms operate and therefore correspond to their functions. Also, it is well-established that a single gene can produce multiple isoforms with varying subcellular localization, which contributes to its functional diversity (Uhlén *et al.* 2015). Inspired by this finding and following the established method in (Chen *et al.* 2021), we validate the predictions of CrossIsoFun by checking its consistency with the subcellular localization of the isoforms.

The subcellular localization of isoforms is extracted from (Uhlén *et al.* 2015), which is predicted from their sequences. Isoforms are then classified into three types: soluble (intracellular), membrane-spanning, or secreted (nonsoluble and nonmembrane). In the experiment, we concentrate on a set of GO terms that are enriched in the interested subcellular locations. “Enriched” means the GO term is overrepresented among genes that encode isoforms found at that location. Seven GO terms are extracted through the rule: GO:0043588 (skin development) as soluble-enriched, GO:0001914 (Regulation of T cell mediated cytotoxicity), GO:0002449 (Lymphocyte mediated immunity), GO:0042102 (Positive regulation of T cell proliferation), GO:0042129 (Regulation of T cell proliferation), and GO:0050671 (Positive regulation of lymphocyte proliferation) as membrane-enriched and GO:0030183 (B cell differentiation) as secreted-enriched. For each chosen GO term, we focus on the MIGs annotated to it, which encode at least one isoform residing in the target location. An isoform is regarded as annotated to the GO term if its prediction score exceeds the mean score across the isoforms of its gene. We use the Jaccard index to measure the concordance between the isoforms associated with the selected GO terms and those located in the corresponding subcellular compartments.

**Figure 2.**
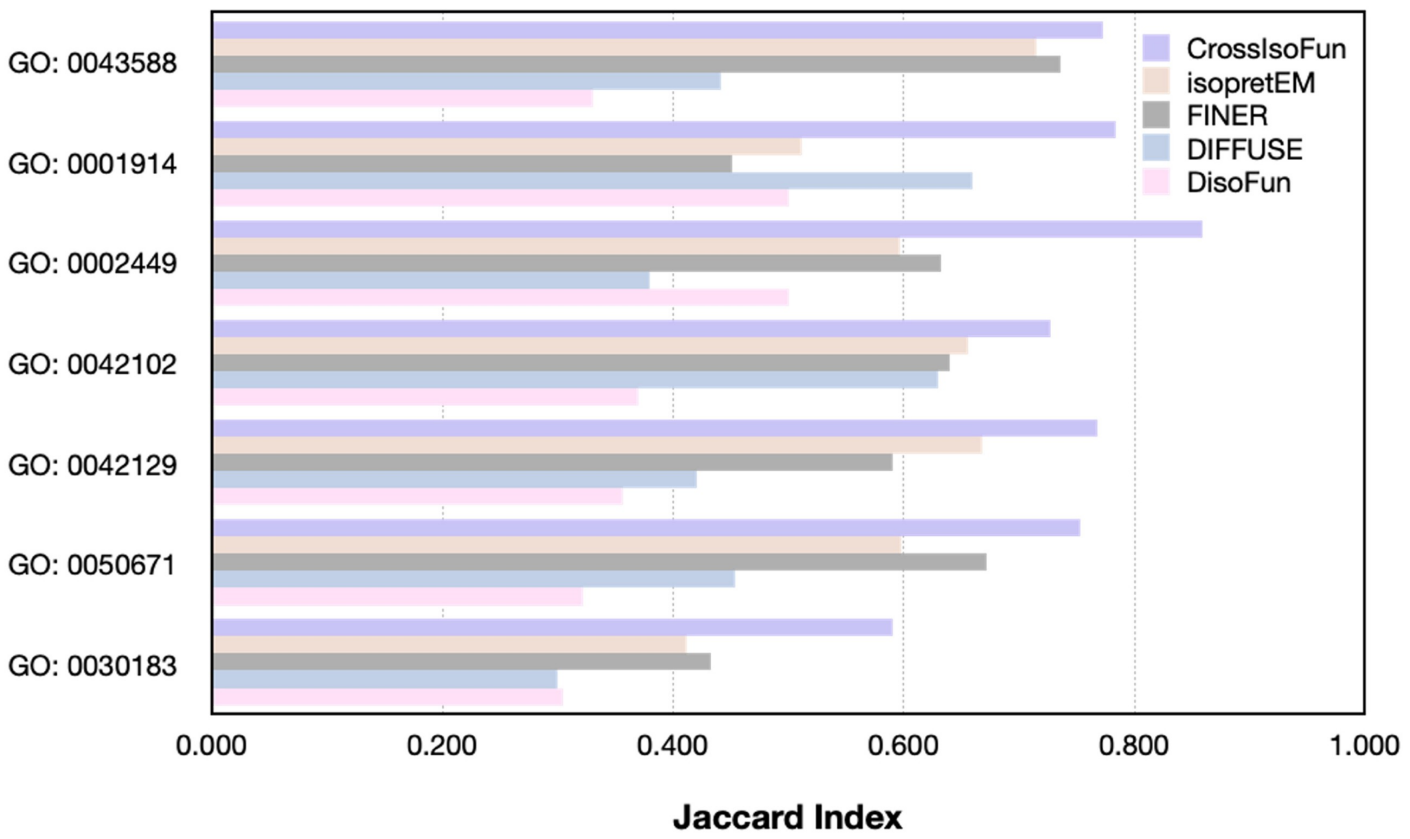
Comparison between CrossIsoFun, isopretEM, FINER, DIFFUSE, and DisoFun in terms of consistency between their predictions on location-enriched GO terms and subcellular localization of the isoforms, where the consistency is measured by the Jaccard index.

As depicted in Fig. 2, CrossIsoFun’s predictions show greater alignment with subcellular localization compared to isopretEM, FINER, DIFFUSE, and DisoFun across all the assessed GO terms. The notable performance differences indicate that localization information inherent in IIIs and sequences might facilitate differentiating isoform functions linked to specific locations. Specifically, IIIs often occur within the same or interconnected cell organelles, and the sequence influences the 3D structures of protein isoforms, thereby impacting their functions and locations within the cell. Moreover, the performance superiority of CrossIsoFun over the other methods underscores the effectiveness of our scheme for incorporating multi-omics data.

### 3.4 Analysis of the effects of model components

To investigate the contribution of each module of CrossIsoFun to its performance, we apply an ablation study by removing the modules one by one and observing how the model performance would be affected. We introduce six variants: (i–iv) CrossIsoFun without VCDN integration, which represents GCNs inputting expression profiles, sequence features, PPIs, and IIIs, respectively; (v) CrossIsoFun without III generation, which means to directly feed expression, sequence, and PPI data to the function prediction module; and (vi) CrossIsoFun without multi-label learning, which refers to performing CrossIsoFun on each GO term separately. We apply these variants and the full model of CrossIsoFun to Dataset A, B, and C. Following the same experimental configuration, we obtain their performance and list it in Supplementary Table S4.

**Figure 3.**
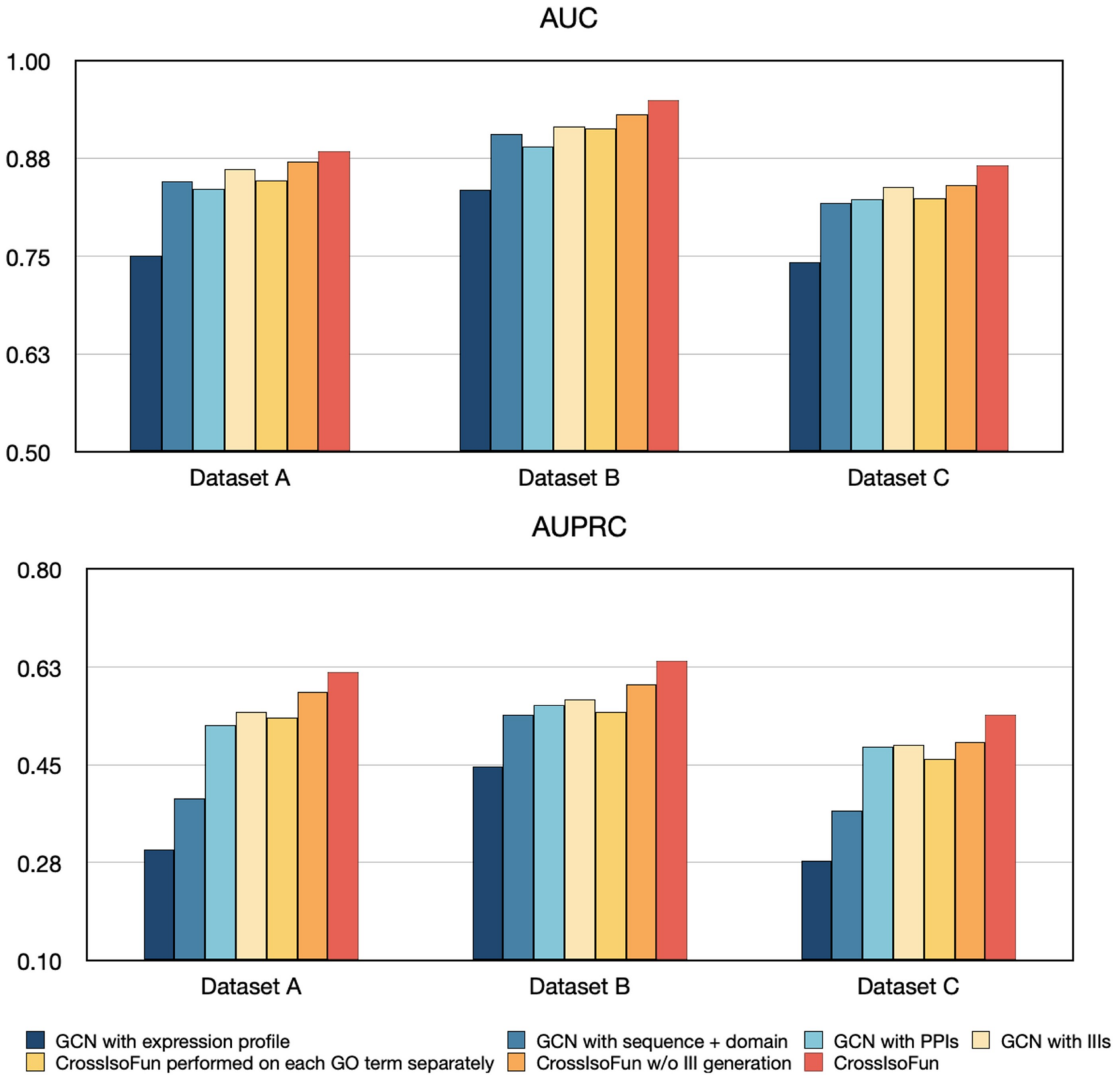
Comparison of prediction performance between variants and CrossIsoFun on Dataset A, B, and C.

Figure 3 shows that CrossIsoFun achieves the best performance (AUC and AUPRC) across all the datasets. For example, the median AUC achieved by CrossIsoFun on Dataset A is 0.884, while the corresponding median AUCs for variants (i)–(vi) are 0.751, 0.845, 0.836, 0.861, 0.847 and 0.870, respectively. The comparison between CrossIsoFun and variants (i)–(iv) reveals that CrossIsoFun largely benefits from incorporating and leveraging the latent functional insights from diverse data types. Adopting a multi-label learning framework (comparing CrossIsoFun and variant (v)) also enhances the model performance, highlighting the need to consider the inter-relationships among different functions. From the results of CrossIsoFun and variant (vi), we observe that although the contribution of the III generation module to AUC improvement is modest to other modules, The AUPRC improvement is quite marked. This indicates the capability of our method in refining the PPIs to IIIs, offering more detailed interactomics insights for isoform function prediction.

We further assess the efficacy of our III generation module by comparing it against several III prediction methods, including IIIDB (Tseng *et al.* 2015), IDMIL-III (Yu *et al.* 2021a,b), DeepIII (Wang *et al.* 2021c), and the III refinement module in FINER. Specifically, for each compared method, we use the same data as CrossIsoFun for model training and III generation. Then the III data is utilized for function prediction with the classification module of CrossIsoFun. Table 3 shows the comparative performance. Our III generation module achieves performance superior to all the others. The average number of isoforms involved in interactions per gene in our generated III network is 3, and the average degree is 12. To validate the accuracy of the generated III data, we collect 56 741 experimentally validated IIIs from the IntAct database, involving isoforms of MIGs in Dataset A. Of these, 2324 interactions are between isoforms in the testing set. In the feature matrix representing the generated III data ($X^{\left(3\right)}$), entries corresponding to these experimentally validated IIIs are set to 1. This updated matrix is then used as input to the prediction module. As shown in Table 3, while the updated III data achieves the best predictive performance, the improvement over the originally generated III data is limited. This indicates that our method generates III data with accuracy comparable to experimentally validated data while providing broader coverage due to its generative capabilities. Our generation module correctly predict 2267 of the 2324 validated IIIs from the testing set (Table 4), outperforming alternative methods. These results further support the effectiveness of our approach in refining III data from PPIs.

**Table 3.** Comparison of predictive performance on Dataset A with III data derived from different III prediction/generation methods.

Method	IIIDB	IDMIL-III	DeepIII	III refinement module in FINER	III generation module in CrossIsoFun	Updated III data
AUC	0.852	0.859	0.862	0.878	0.884	**0.891**
AUPRC	0.523	0.541	0.557	0.604	0.616	**0.623**

Bold values indicate the maximal AUC or AUPRC achieved with III data derived from different III prediction/generation methods.

**Table 4.** Comparison of the number of validated IIIs correctly predicted by III prediction/generation methods.

Method	IIIDB	IDMIL-III	DeepIII	III refinement module in FINER	III generation module in CrossIsoFun
Correctly predicted IIIs	1618	1767	1829	2201	**2267**

Bold values indicate the maximal number of validated IIIs correctly predicted by different III prediction/generation methods.

### 3.5 Case study

To assess the ability of CrossIsoFun to differentiate isoform functions for genes, we utilize genes with isoform-level functional annotations, as documented in (Shaw *et al.* 2019) and (Qiu *et al.* 2022). Eight genes with four functions annotated to them are extracted: ACE, ACMSD, and GCH1 are annotated with “metal ion binding” (GO:0046872); ADK, AIFM1, and PPP1R8 are annotated with “nucleus” (GO:0005634); RPL13 and MCM3 are annotated with “structural constituent of ribosome” (GO:0003735) and “ATP hydrolysis activity” (GO:0016887), respectively.

CrossIsoFun accurately distinguishes 16 out of the 18 GO annotations, achieving an 88.9% accuracy. The performance surpasses that of IsofunGO, FINER, IsoFrog, DMIL-IsoFun, IsoResolve, DIFFUSE, and DisoFun, highlighting the enhanced precision of CrossIsoFun in recognizing isoform functions. The details of the prediction results are shown in Supplementary Table S5.

We conduct additional experiments with CrossIsoFun and isopretEM. For this, we utilize a larger dataset comprising 307 isoform-level functional annotations from (Karlebach *et al.* 2023). This dataset includes 149 isoforms from 62 genes annotated with 97 GO terms. CrossIsoFun achieves a higher AUC (0.68) compared to isopretEM (0.63) and predicts more GO terms (145 versus 108). These results underscore the enhanced capability of CrossIsoFun in isoform function prediction.

## 4 Conclusion

In this article, we propose CrossIsoFun, a multi-omics integrative method for isoform function prediction. Its key contributions are: (i) combining omics-specialized learning and multi-omics integration within label space, thereby addressing the limitations of current methods in capturing inter-omics relationships and label-level insights. (ii) proposing a cycleGAN-structured framework to generate IIIs from expression profiles, sequence features, and PPIs, enriching the interactomics data and enhancing the understanding of isoform functions. Its algorithmic innovation lies in the novel integration of these components for isoform function prediction: (i) III Generation Module: Our framework introduces a multi-input, multi-output cycleGAN architecture to generate III data, addressing the scarcity of such data. This module leverages auxiliary objectives to extract and integrate information from different omics data sources, enhancing the model’s ability to generalize across datasets. (ii) Modified VCDN Architecture: We adapt the VCDN framework to multi-label prediction tasks, enabling it to effectively capture cross-view correlations among data modalities, model dependencies between functional labels, and address label imbalance issues. These advancements allow our framework to bridge the gaps in III data and provide robust predictions for isoform functions.

We perform train-test split experiments on three tissue-naive and 15 tissue-specific datasets. CrossIsoFun outperforms the state-of-the-art methods including isopretEM, isofunGO, FINER, IsoFrog, DMIL-IsoFun, IsoResolve, DIFFUSE, DisoFun, IsoFun, WLRM, iMILP, and mi-SVM across all the datasets. An in-depth analysis of the results from tissue-specific datasets shows consistency between the predictions and the subcellular localization of the isoforms, validating CrossIsoFun’s capability to differentiate the functions of isoforms within different locations. In the ablation experiment, CrossIsoFun achieves better performance than any other combination of its modules. Finally, we demonstrate the effectiveness of our method in distinguishing the isoform functions for genes through a case study. Our results show that the strategy of CrossIsoFun to process individual omics data and integrate multi-omics information significantly enhances the model performance compared to the previous results.

To address the time efficiency of CrossIsoFun, we evaluate its runtime and compare it to other state-of-the-art methods using Dataset A. All experiments are conducted on a system equipped with a 32-core Intel(R) Xeon(R) Gold 5218 CPU @2.30 GHz and an NVIDIA Tesla V100S PCIe 32GB GPU. CrossIsoFun requires 1472.5 min to process Dataset A. Comparatively, the runtime of other methods is as follows: 507.3 (isopretEM), 10.7 (IsofunGO), 903.4 (FINER), 5203.2 (IsoFrog), 522.9 (DMIL-IsoFun), 208.3 (IsoResolve), 237.4 (DIFFUSE), 735.3 (IsoFun), 897.7 (iMILP), 12 595.2 (DisoFun), 310.4 (WLRM), and 622.8 (mi-SVM) minutes, respectively. CrossIsoFun demonstrates faster execution than IsoFrog and DisoFun but is slower than other methods. This is primarily due to its deep learning-based approach, which requires extensive training epochs for III generation and isoform function prediction. To address this, we plan to optimize the training process and explore more computationally efficient architectures in future work.

Our method has the potential for improvement in several ways. First, CrossIsoFun refines IIIs from PPIs by integrating expression and sequence data. However, our method does not utilize data from existing III databases. Despite their nonexhaustive nature, incorporating these III data could potentially yield more accurate III inferences and thereby improve isoform function prediction. Moreover, CrossIsoFun focuses primarily on specific types of data. Integrating a broader range of omics data, such as epigenomics, diseasomics, and metabolomics, could offer more comprehensive insights. Given the complexity of deep learning models, fine-tuning the existing model architecture with more efficient structures, like Transformers or variants of GCNs, might also enhance both predictive accuracy and processing speed. We recognize that isoforms from the same gene often interact and complement each other to perform complex biological functions. Future work will focus on developing advanced models that explicitly account for these cooperative behaviors, moving beyond the current framework of maximum score representation. Finally, in our framework, VCDN integrates predictions from III, sequence, and expression data into a cross-omics discovery tensor (n×n×n, n represents the number of GO labels). The tensor size increases significantly with larger GO label sets, making computations intensive. To address this, we aim to explore efficient sampling strategies, scalable machine learning frameworks, and advanced optimization techniques in future work.

## Supplementary data

Supplementary data are available at *Bioinformatics* online.

Conflict of interest: None declared.

## Funding

This work was supported by the STI2030-Major Projects (No. 2022ZD0213700); the National Natural Science Foundation of China [62350004, 62332020]; and the Project of Xiangjiang Laboratory (No. 23XJ01011). This work was carried out in part using computing resources at the High-Performance Computing Center of Central South University.

## Supplementary Material

btae742_Supplementary_Data

## Data Availability

The data underlying this article are publicly available with their sources described in the article.
