# Supplementary material for: CrossIsoFun: predicting isoform functions using the integration of multi-omics data
Source: Bioinformatics. 2024 Dec 16;41(1):btae742. doi: 10.1093/bioinformatics/btae742 (PMC11706537; doi:10.1093/bioinformatics/btae742)
Supplement: btae742_Supplementary_Data [file btae742_supplementary_data.docx]

*Supplementary materials for*

**CrossIsoFun: predicting isoform functions using the integration of multi-omics data**

***List of supplementary content:***

| **Supplementary Note S1** | The data collection and preprocessing of Dataset B and C. |
| --- | --- |
| **Supplementary Note S2** | The loss functions for auxiliary training objectives of the autoencoder in the cycleGAN-structured III generation model. |
| **Supplementary Note S3** | The procedure of symmetrizing the III generation result. |
| **Supplementary Note S4** | The training and testing of the omics-specific GCNs. |
| **Supplementary Figure S1** | Prediction performance of CrossIsoFun on GO terms with different sizes and categories (based on Dataset A). |
| **Supplementary Table S1** | PPI databases utilized in the experiments of CrossIsoFun on tissue-naïve datasets with corresponding References and URLs. |
| **Supplementary Table S2** | The hyperparameters of CrossIsoFun used in the experiments. |
| **Supplementary Table S3** | The performance of CrossIsoFun on GO terms specific to each tissue. |
| **Supplementary Table S4** | The prediction performance of variants and CrossIsoFun in the ablation experiment. |
| **Supplementary Table S5** | Literature support for 18 isoforms of 8 genes on four GO terms. |

**Supplementary Note S1.** The data collection and preprocessing of Dataset B and C.

Dataset B initially includes expression data for 24,274 isoforms of 19,201 mouse genes from 116 SRA studies, consisting of 365 RNA-seq experiments. We search CDS in the RefSeq database (GRCm39), excluding isoforms without CDS annotations. Genes of these isoforms and the other isoforms of the genes are also dropped. We then retain 20,118 isoforms from 15,850 genes (13,067 SIGs and 2,783 MIGs). The methods for preprocessing sequence features and PPIs are identical to the ones in Dataset A.

Dataset C contains expression data from 456 RNA-seq experiments across 29 human full-length isoform sequencing studies in the NCBI SRA database. The CDS, conserved domains, and PPIs of isoforms are collected and processed similarly to Dataset A and B, yielding multi-omics data for 32,983 isoforms from 18,712 genes (11,633 SIGs and 7,079 MIGs).

**Supplementary Note S2.** The loss functions for auxiliary training objectives of the autoencoder in the cycleGAN-structured III generative model [1].

The training of the autoencoder incorporates auxiliary objectives to enhance its integration and generalization capabilities, which include:

1. **Generating Expression Profiles**: involves the generation of expression profiles from sequence features and PPIs.
2. **Generating Sequence Features**: focuses on generating sequence features from expression profiles and PPIs.
3. **Generating III Data:** entails generating IIIs from expression profiles and sequence features, different from generating III data from expression, sequence, and PPI data in the main text.

For each of the objectives, we calculate its generation loss, reconstruction loss, and cycle consistency loss, paralleling the loss calculations for the primary objective described in the main text of the paper. The specifics are given in the following sections.

The generation and reconstruction losses for the objective (1) **Generating Expression Profiles** can be calculated as:

| $L_{GEN}^{\left( 1 \right)}=\sum_{i=1}^{n} L_{MSE}(\boldsymbol{x}_{i}^{\left( 1 \right)},\boldsymbol{G}^{\left( 1 \right)}\left( \boldsymbol{E}\left( \boldsymbol{x}_{i}^{\left( 2 \right)},\boldsymbol{x}_{i}^{\left( 3 \right)} \right) \right))$ | (1) |
| --- | --- |
| $L_{REC}^{\left( 1 \right)} = \sum_{i=1}^{n} L_{MSE}(\boldsymbol{x}_{i}^{\left( 2 \right)},\boldsymbol{G}^{\left( 2 \right)}\left( \boldsymbol{E}\left( \boldsymbol{x}_{i}^{\left( 2 \right)},\boldsymbol{x}_{i}^{\left( 3 \right)} \right) \right)) + \sum_{i=1}^{n} L_{BCE}(\boldsymbol{x}_{i}^{\left( 3 \right)},\boldsymbol{G}^{\left( 3 \right)}\left( \boldsymbol{E}\left( \boldsymbol{x}_{i}^{\left( 2 \right)},\boldsymbol{x}_{i}^{\left( 3 \right)} \right) \right))$ | (2) |

The cycle consistency loss is utilized to ensure that the generated and reconstructed data can be employed for further generation and reconstruction via the autoencoder:

| $L_{cycle}^{(1)}=\sum_{k=1}^{3} \sum_{i=1}^{n} L1(\boldsymbol{x}^{\left( k \right)}, \boldsymbol{G}^{\left( k \right)}\boldsymbol{(E}\left( \boldsymbol{G}^{\left( 2 \right)}\left( \boldsymbol{E}\left( \boldsymbol{x}_{i}^{\left( 2 \right)},\boldsymbol{x}_{i}^{\left( 3 \right)} \right) \right), \boldsymbol{G}^{\left( 3 \right)}\left( \boldsymbol{E}\left( \boldsymbol{x}_{i}^{\left( 2 \right)},\boldsymbol{x}_{i}^{\left( 3 \right)} \right) \right) \right))$ | (3) |
| --- | --- |

Then the total loss for objective (1) is

| $L_{AE}^{(1)}=\lambda_{1}L_{GEN}^{(1)}$ + $\lambda_{2}L_{REC}^{(1)}$ + $\lambda_{3}L_{cycle}^{(1)}$ | (4) |
| --- | --- |

Similarly, the functions for calculating losses for the objective (2) **Generating Sequence Features** are:

| $L_{GEN}^{\left( 2 \right)}=\sum_{i=1}^{n} L_{MSE}(\boldsymbol{x}_{i}^{\left( 2 \right)},\boldsymbol{G}^{\left( 2 \right)}\left( \boldsymbol{E}\left( \boldsymbol{x}_{i}^{\left( 1 \right)},\boldsymbol{x}_{i}^{\left( 3 \right)} \right) \right))$ | (5) |
| --- | --- |
| $L_{REC}^{\left( 2 \right)} = \sum_{i=1}^{n} L_{MSE}(\boldsymbol{x}_{i}^{\left( 1 \right)},\boldsymbol{G}^{\left( 1 \right)}\left( \boldsymbol{E}\left( \boldsymbol{x}_{i}^{\left( 1 \right)},\boldsymbol{x}_{i}^{\left( 3 \right)} \right) \right)) + \sum_{i=1}^{n} L_{BCE}(\boldsymbol{x}_{i}^{\left( 3 \right)},\boldsymbol{G}^{\left( 3 \right)}\left( \boldsymbol{E}\left( \boldsymbol{x}_{i}^{\left( 1 \right)},\boldsymbol{x}_{i}^{\left( 3 \right)} \right) \right))$ | (6) |
| $L_{cycle}^{(2)}=\sum_{k=1}^{3} \sum_{i=1}^{n} L1(\boldsymbol{x}^{\left( k \right)}, \boldsymbol{G}^{\left( k \right)}\boldsymbol{(E}\left( \boldsymbol{G}^{\left( 1 \right)}\left( \boldsymbol{E}\left( \boldsymbol{x}_{i}^{\left( 1 \right)},\boldsymbol{x}_{i}^{\left( 3 \right)} \right) \right), \boldsymbol{G}^{\left( 3 \right)}\left( \boldsymbol{E}\left( \boldsymbol{x}_{i}^{\left( 1 \right)},\boldsymbol{x}_{i}^{\left( 3 \right)} \right) \right) \right))$ | (7) |
| $L_{AE}^{(2)}=\lambda_{1}L_{GEN}^{(2)}$ + $\lambda_{2}L_{REC}^{(2)}$ + $\lambda_{3}L_{cycle}^{(2)}$ | (8) |

For objective (3) **Generating III Data**, the losses are calculated as below:

| $L_{GEN}^{\left( 3 \right)}=\sum_{i=1}^{n} L_{BCE}(\boldsymbol{x}_{i}^{\left( 3 \right)},\boldsymbol{G}^{\left( 3 \right)}\left( \boldsymbol{E}\left( \boldsymbol{x}_{i}^{\left( 1 \right)},\boldsymbol{x}_{i}^{\left( 2 \right)} \right) \right))$ | (9) |
| --- | --- |
| $L_{REC}^{\left( 3 \right)} = \sum_{k=1}^{2} \sum_{i=1}^{n} L_{MSE}(\boldsymbol{x}_{i}^{\left( k \right)},\boldsymbol{G}^{\left( k \right)}\left( \boldsymbol{E}\left( \boldsymbol{x}_{i}^{\left( 1 \right)},\boldsymbol{x}_{i}^{\left( 2 \right)} \right) \right)))$ | (10) |
| $L_{cycle}^{(2)}=\sum_{k=1}^{3} \sum_{i=1}^{n} L1(\boldsymbol{x}^{\left( k \right)}, \boldsymbol{G}^{\left( k \right)}\boldsymbol{(E}\left( \boldsymbol{G}^{\left( 1 \right)}\left( \boldsymbol{E}\left( \boldsymbol{x}_{i}^{\left( 1 \right)},\boldsymbol{x}_{i}^{\left( 2 \right)} \right) \right), \boldsymbol{G}^{\left( 2 \right)}\left( \boldsymbol{E}\left( \boldsymbol{x}_{i}^{\left( 1 \right)},\boldsymbol{x}_{i}^{\left( 2 \right)} \right) \right) \right))$ | (11) |
| $L_{AE}^{(3)}=\lambda_{1}L_{GEN}^{(3)}$ + $\lambda_{2}L_{REC}^{(3)}$ + $\lambda_{3}L_{cycle}^{(3)}$ | (12) |
|  |  |

**Supplementary Note S3.** The procedure of symmetrizing the III generation result.

The generative model in CrossIsoFun produces an III vector for each isoform using its expression, sequence, and PPI features. However, it doesn't account for the symmetry in the interactions among different isoforms during this process. As a result, the matrix representing the III generation output for all the isoforms might be asymmetric, being unsuitable for direct use as the interactome input for isoform function prediction. Therefore, we perform symmetrization on the generated matrix to make it align with the symmetric nature of biological interactions and appropriate for subsequent analyses in isoform function prediction. The details of the symmetrization process are as follows:

**Identification of Asymmetric Entries**: In the generated matrix representing IIIs, we identify the symmetric entry corresponding to each entry (value). The symmetric entry of a value at position (*i*, *j*) is the value at position (*j*, *i*).

**Adjustment for Values Greater than 0.5**: For every entry in the matrix that is greater than 0.5, we compare it with its symmetric counterpart. The higher value between the two is then assigned to both positions. This step ensures that strong interactions (values > 0.5) are consistently represented in both directions, maintaining the integrity of significant interactions.

**Processing Values Less than 0.5**: Then, for entries that are less than 0.5, whose symmetric counterparts are also less than 0.5, we compute the average of these two values. Both the entry and its symmetric counterpart are then assigned with this average value. This step is crucial for maintaining consistency in weaker interactions, ensuring that they are equally represented in both directions.

**Resulting Symmetric III matrix**: Following these adjustments, the generated III data is transformed into a symmetric matrix. This symmetric matrix is now more representative of the true interactome structure and is better suited as an interactomics input for isoform function prediction models.

**Supplementary Note S4.** The training and testing of the omics-specific GCNs [2].

During the training phase, the GCN for each omics data is optimized utilizing the feature matrix of the training samples **X**_tr_ $\boldsymbol{\in}\mathbb{R}^{n_{tr}\times d}$and and the corresponding adjacency matrix **A**_tr_ $\boldsymbol{\in}\mathbb{R}^{n_{tr}\times n_{tr}}$. The predictions on the training set are given by ${\hat{\mathbf{Y}}}_{tr}\boldsymbol{\in}\mathbb{R}^{n_{tr}\times T}= GCN\left( \mathbf{X}_{tr}, \mathbf{A}_{tr} \right)$. The GCN employs WBCE Loss (detailed in the main text) to evaluate the predicted probabilities against the actual labels of the training samples:

| $L_{GCN}=\sum_{m=1}^{g_{tr}} L_{WBCE}\left( {\hat{\boldsymbol{y}}}_{m},\boldsymbol{y}_{m} \right),$ | (13) |
| --- | --- |

where $\boldsymbol{y}_{m}$ represents the one-hot encoded label vector of the *m*th gene in the training samples, which is the *m*th row of $\boldsymbol{Y}_{tr}$, and ${\hat{\boldsymbol{y}}}_{m}$ is the predicted result of the corresponding gene. Here, ⊙ denotes element-wise multiplication, and log is the element-wise natural logarithm. Additionally, to address label imbalance in the training set, we assign different weights to the losses for different classes, which is achieved by $w_{m,t}$ in Equation (13). Specifically, $w_{m,t}$ is set as the inverse of the occurrence rate of the label corresponding to the *m*th sample for the GO term *t* in the training dataset.

In the testing phase, to acquire the input of GCN, first, we concatenate **X**_tr_ with the test data **X**_te_ $\boldsymbol{\in}\mathbb{R}^{n_{te}\times d}$, yielding **X**_trte_ =$\left[ \begin{matrix} \mathbf{X}_{\mathrm{tr}} \\ \mathbf{X}_{\mathrm{te}} \end{matrix} \right]$ $\boldsymbol{\in}\mathbb{R}^{n_{trte}\times d}$, where $n_{trte}= n_{tr}+n_{te}$. Then the adjacency matrix **A**_trte_ $\boldsymbol{\in}\mathbb{R}^{n_{trte}\times n_{trte}}$ corresponding to **X**_trte_ is obtained with Equation (8) in the main text of the paper. However, only the entries representing the relationships between training and testing samples are retained, while others are set as 0. Utilizing the trained GCN model GCN(⋅), the prediction for **X**_trte_ and **A**_trte_ is obtained: ${\hat{\mathbf{Y}}}_{trte}\boldsymbol{\in}\mathbb{R}^{n_{trte}\times T}= GCN\left( \mathbf{X}_{trte}, \mathbf{A}_{trte} \right)$. The predicted label probability distribution for the testing sample is the last $n_{te}$ rows of ${\hat{\mathbf{Y}}}_{trte}$. This approach leverages the features of test samples and their correlations with the training samples for accurate function prediction.


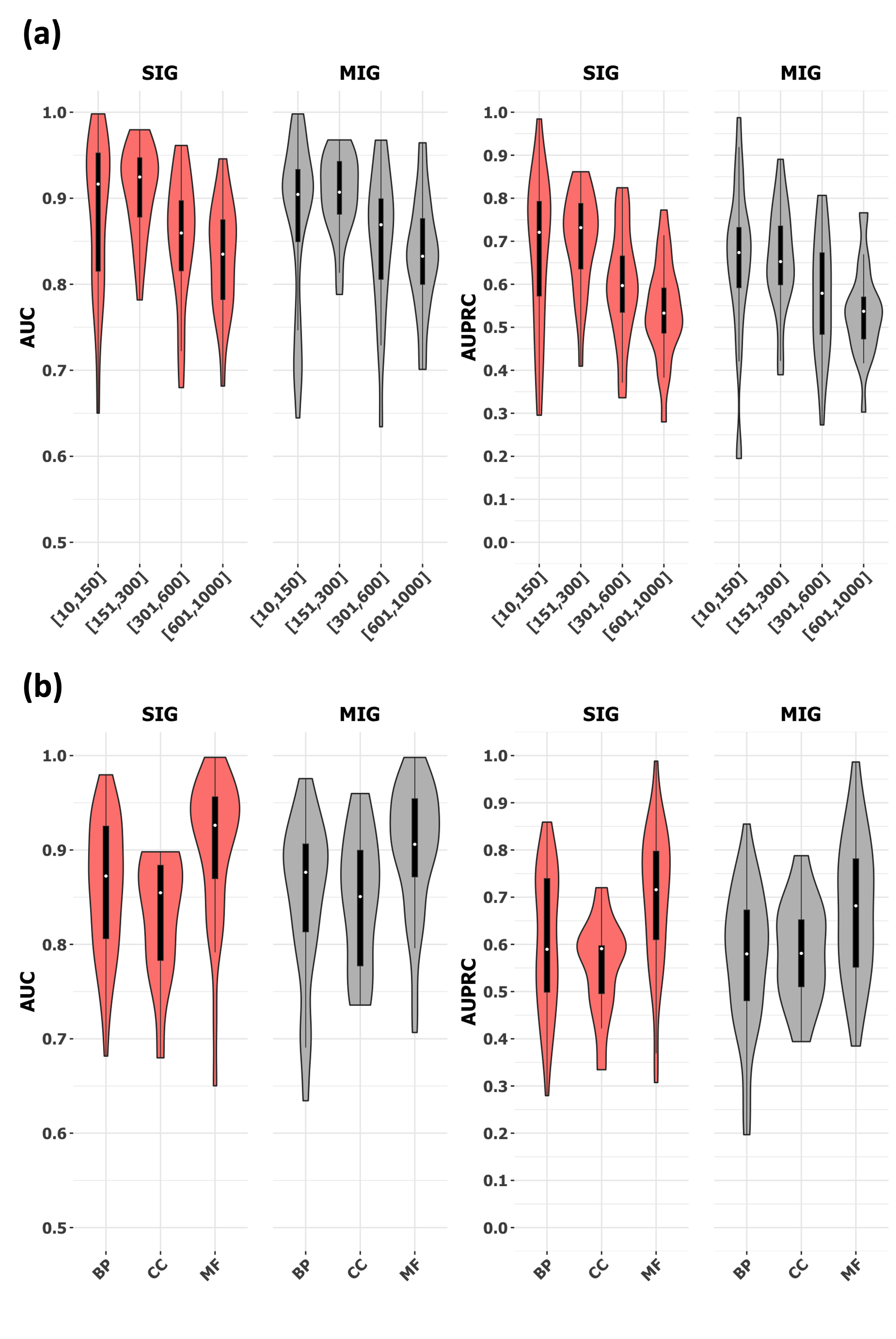


**Figure S1.** Prediction performance of CrossIsoFun on GO terms with different sizes and categories (based on Dataset A).

**Table S1**. PPI databases utilized in the experiments of CrossIsoFun on tissue-naïve datasets with corresponding references and URLs.

| PPI databases | References | URLs |
| --- | --- | --- |
| IntAct | Hermjakob et al., 2004 [3] | https://www.ebi.ac.uk/intact/ |
| MINT | Chatr-Aryamontri et al., 2007 [4] | https://mint.bio.uniroma2.it/ |
| BioGRID | Oughtred et al., 2019 [5] | https://thebiogrid.org/ |
| CORUM | Giurgiu et al., 2019 [6] | https://mips.helmholtz-muenchen.de/corum/ |
| TRANSFAC | Matys et al., 2003 [7] | https://genexplain.com/transfac/ |
| HURI | Luck et al., 2020 [8] | http://www.interactome-atlas.org/ |
| STRING | Szklarczyk et al., 2019 [9] | https://string-db.org/ |
| HPRD | Keshava Prasad, T. et al. 2009 [10] | http://www.hprd.org/ |
| primeKG | Chandak et al., 2023 [11] | https://zitniklab.hms.harvard.edu/projects/PrimeKG/ |

**Table S2**. The hyperparameters of CrossIsoFun used in the experiments.

| parameter | λ1 | λ2 | λ3 | a |
| --- | --- | --- | --- | --- |
| value | 1 | 1 | 0.05 | 5 |

* In our computational experiments, the Adam optimizer [12] is used to optimize the model. The sizes of the autoencoder interlayers are 2048, 1024, 256, 1024, and 2048 respectively. The feature dimensions of the convolutional layers in GCNs are 400, 200, and 200 respectively. The output dimension of the input layer of VCDN is 512.

**Table S3**. The performance of CrossIsoFun on GO terms specific to each tissue.

| BTO_id | Tissue | # of GO terms | AUC | AUPRC |
| --- | --- | --- | --- | --- |
| Major tissue (12) | | | | |
| BTO_0000141 | bone marrow | 3 | 0.996 | 0.993 |
| BTO_0000562 | heart | 131 | 0.983 | 0.872 |
| BTO_0000648 | intestine | 6 | 0.995 | 0.995 |
| BTO_0000763 | lung | 7 | 0.996 | 0.970 |
| BTO_0000775 | lymphocyte | 165 | 0.965 | 0.829 |
| BTO_0001078 | placenta | 8 | 0.874 | 0.766 |
| BTO_0001103 | skeletal muscle | 49 | 0.956 | 0.804 |
| BTO_0001253 | skin | 29 | 0.980 | 0.875 |
| BTO_0001363 | testis | 4 | 0.997 | 0.976 |
| BTO_0001379 | thyroid gland | 3 | 0.999 | 0.993 |
| BTO_0001422 | uterine endometrium | 3 | 0.995 | 0.951 |
| BTO_0001487 | adipose tissue | 13 | 0.990 | 0.929 |
| Brain tissue (3) | | | | |
| BTO_0000232 | cerebellum | 14 | 0.979 | 0.939 |
| BTO_0001279 | spinal cord | 29 | 0.981 | 0.901 |
| BTO_0000233 | cerebral cortex | 7 | 0.927 | 0.817 |

**Table S4**. The prediction performance of combinations of CrossIsoFun modules and CrossIsoFun in the ablation experiment.

| Module combination | GCN with expr | GCN with sequence+ domain | GCN with PPI | GCN with III | CrossIsoFun on each GO term | CrossIsoFun w/o III generation | CrossIsoFun |
| --- | --- | --- | --- | --- | --- | --- | --- |
| AUC | | | | | | | |
| Dataset A | 0.751 | 0.845 | 0.836 | 0.861 | 0.847 | 0.870 | 0.884 |
| Dataset B | 0.834 | 0.906 | 0.889 | 0.916 | 0.913 | 0.931 | 0.949 |
| Dataset C | 0.742 | 0.818 | 0.822 | 0.838 | 0.824 | 0.841 | 0.865 |
| AUPRC | | | | | | | |
| Dataset A | 0.299 | 0.391 | 0.521 | 0.545 | 0.534 | 0.581 | 0.616 |
| Dataset B | 0.448 | 0.539 | 0.558 | 0.567 | 0.545 | 0.595 | 0.637 |
| Dataset C | 0.278 | 0.369 | 0.483 | 0.486 | 0.461 | 0.491 | 0.539 |

**Table S5**. Literature support for 18 isoforms of 8 genes on four GO terms.


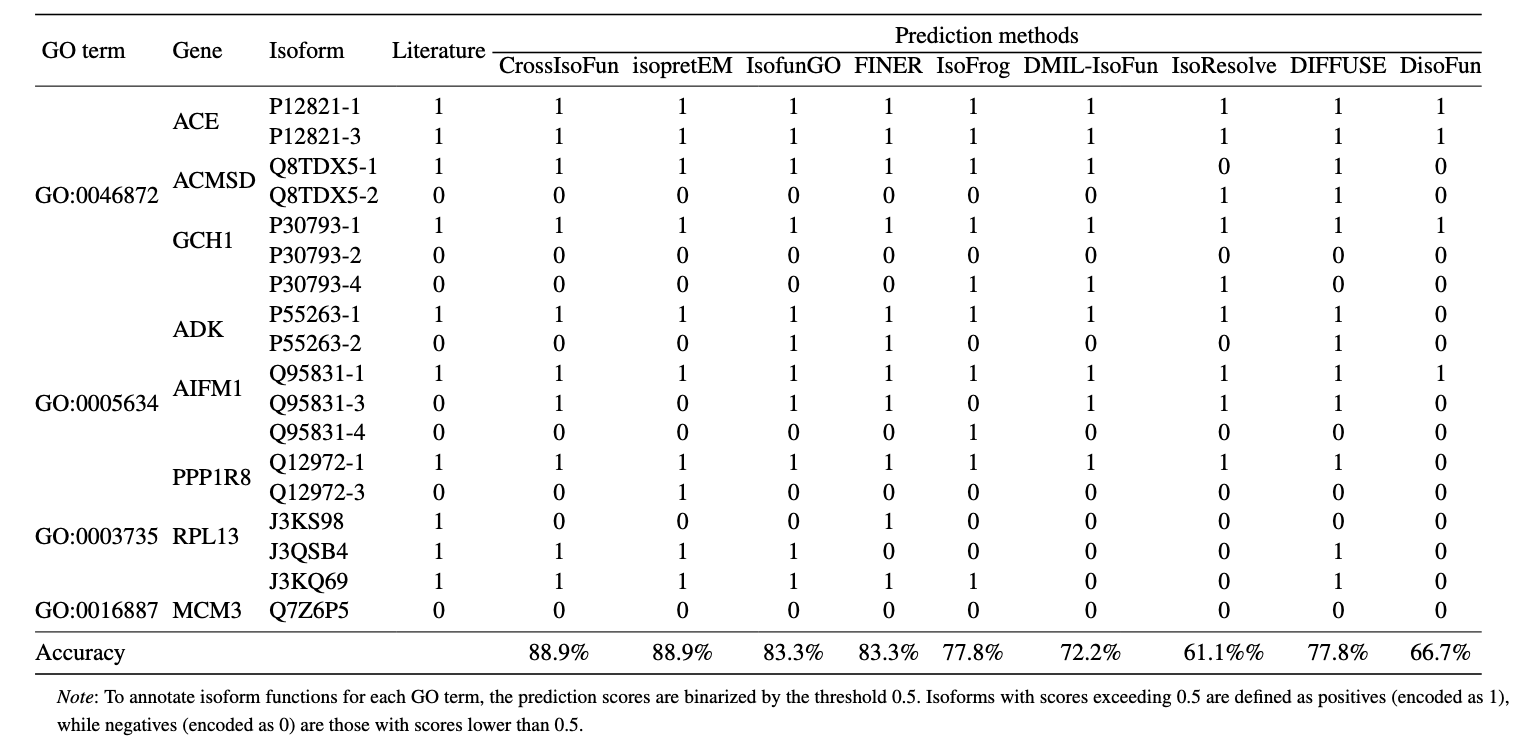


**References:**

1. Wang, Q. et al. (2021a). Generative partial multi-view clustering with adaptive fusion and cycle consistency. IEEE Transactions on Image Processing, 30, 1771–1783.
2. Wang, T. et al. (2021b). Mogonet integrates multi-omics data using graph convolutional networks allowing patient classification and biomarker identification. Nature Communications, 12(1), 3445.
3. Hermjakob, H. et al. (2004) Intact: an open source molecular interaction database. Nucleic acids research, 32(supply_1), D452–D455.
4. Chatr-Aryamontri, A. et al. (2007) Mint: the molecular interaction database. Nucleic acids research, 35, D572–D574.
5. Oughtred, R. et al. (2019) The biogrid interaction database: 2019 update. Nucleic acids research, 47(D1), D529–D541.
6. Giurgiu, M. et al. (2019) Corum: the comprehensive resource of mammalian protein complexes—2019. Nucleic acids research, 47(D1), D559–D563.
7. Matys, V. et al. (2003) TRANSFAC ® : transcriptional regulation, from patterns to profiles . Nucleic Acids Research, 31(1), 374–378.
8. Luck, K. et al. (2020) A reference map of the human binary protein interactome. Nature, 580(7803), 402–408.
9. Szklarczyk, D. et al. (2019) String v11: protein–protein association networks with increased coverage, supporting functional discovery in genome-wide experimental datasets. Nucleic acids research, 47(D1), D607–D613.
10. Keshava Prasad, T. et al. (2009) Human protein reference database—2009 update. Nucleic acids research, 37(supply_1), D767–D772.
11. Chandak, P. et al. (2023) Building a knowledge graph to enable precision medicine. Scientific Data, 10(1), 67.
12. Kingma D.P. et al. (2014) Adam: A method for stochastic optimization. arXiv preprint arXiv:1412.6980.
